# S116. THE IMPACT OF PSYCHOTIC EXPERIENCES IN THE EARLY STAGES OF MENTAL HEALTH PROBLEMS IN YOUNG PEOPLE

**DOI:** 10.1093/schbul/sby018.903

**Published:** 2018-04-01

**Authors:** Kareen Heinze, Ashleigh Lin, Barnaby Nelson, Renate Reniers, Rachel Upthegrove, Latoya Clarke, Ayesha Roche, Lowrie Angelique, Stephen Wood

**Affiliations:** 1 University of Birmingham; 2 Telethon Kids Institute; 3 Orygen, The National Centre of Excellence in Youth Mental Health; 4 University of Warwick; 5 University of Sheffield

## Abstract

**Background:**

Anxiety and depressive symptoms and psychotic experiences constitute common features of emerging mental disorders in young people. Psychotic experiences and the ultra-high risk (UHR) state for psychosis appear to have a particular importance for clinical presentation, progression of symptomatology, quality of life and functioning, but the impact of psychotic experiences in individuals seeking help at non-UHR services, compared to UHR services, is under-researched.

**Methods:**

69 young people (Mage ± SD at baseline = 20.8 ± 2.6, range 16–26 years, 48 females) presenting to mental health services were grouped according to UHR and non-UHR status. They were assessed at baseline for psychotic experiences, anxiety and depressive symptoms, psychological distress, psychosocial functioning and quality of life. They were followed up at three, six, and 12 months. Data were analysed using mixed and general linear modelling.

**Results:**

UHR individuals reported higher levels of depressive symptoms and lower levels of role functioning at baseline compared to non-UHR individuals. Significant differences were evident over time for psychological distress and quality of life, with greater impairment developing in those at UHR. No robust differences were reported for anxiety symptoms or social functioning.

**Discussion:**

Psychotic experiences appear to be particularly associated with depressive symptoms and psychological distress, impaired role functioning and quality of life in help-seeking young people in the medium-term. It is therefore important to pay special attention to psychotic experiences in the early stages of mental health problems even if psychotic symptoms are not the main motivation for help-seeking.

